# Effect of dietary supplementation of plantain herb, lemongrass and their combination on milk yield, immunity, liver enzymes, serum, and milk mineral status in dairy cows

**DOI:** 10.5455/javar.2024.k764

**Published:** 2024-03-31

**Authors:** Md. Aliar Rahman, Md. Rahat Ahmad Redoy, Rakhi Chowdhury, Mohammad Al-Mamun

**Affiliations:** Department of Animal Nutrition, Bangladesh Agricultural University, Mymensingh, Bangladesh

**Keywords:** Dairy cows, plantain, lemongrass, immunity, liver enzymes, milk yield, zinc

## Abstract

**Objective:**

This research aimed to assess the effects of dried plantain herb, lemongrass, and their combination on milk yield, immunological, liver enzymatic, serum, and milk mineral status in dairy cows.

**Materials and Methods:**

Twenty cows were arbitrarily assigned to 4 diets. Cows were given a basal ration considered as control diet (CL-D) having 14.93% crude protein (CP)and 10.96 MJ ME per kg dry matter (DM). Each cow was given 100 gm plantain, 100 gm lemongrass, and 50 gm plantain + 50 gm lemongrass with CL-D and taken as plantain diet (PT-D), lemongrass diet (LG-D), and plantain-lemongrass diet (PL-D), daily for 63 days, respectively. Blood and milk samples were taken four times at an interval of 14 days. Data were analyzed using a two-way repeated measures analysis of covariance.

**Results:**

Better DM consumption and milk yield were observed in the PT-D and LG-D compared to the CL-D (*p ≤ 0*.05). LG-D improved the milk’s total solids, protein, and fat compared to CL-D (*p <* 0.05). Substantially, herbal groups improved serum albumin and reduced globulin concentrations compared to CL-D. LG-D had the highest serum immunoglobulin G, while herbal groups effectively reduced the liver enzymes compared to CL-D. Herbal groups did not affect serum and milk’s calcium and phosphorus concentrations, while LG-D and PL-D substantially improved serum and milk zinc concentrations.

**Conclusions:**

Both plantain and lemongrass improved dairy cows’ DM consumption and milk yield. Plantain and/or lemongrass enhanced the immune system and liver health, but not serum and milk calcium and phosphorus level. Lemongrass and a combination of plantain and lemongrass increased the serum and milk zinc concentrations.

## Introduction

The major goal of the dairy industry is to improve cows’ health, milk yield, and quality. However, dairy cows frequently grow under substandard conditions in terms of housing, management, nutrition, and excessive heat or cold [1]. In addition, dairy cows often face sub-clinical and clinical diseases, especially mastitis. These situations cause animals to produce more reactive oxygen species (ROS), damaging cells and ultimately resulting in oxidative stress (OS). The imbalance of ROS and antioxidants is called OS, which impairs health, milk yield, and quality in dairy cows [2]. However, various enzymatic and non-enzymatic mechanisms are directly associated with preventing OS. The non-enzymatic mechanism involves the inclusion of dietary antioxidant-rich additives, which are ridiculous in vitamins A and E, minerals, especially selenium, zinc, and polyphenols [3].

Moreover, the inclusion of antioxidant-rich herbs might be suitable aspirants to prevent OS and improve the health status, immunity, and product quality of dairy animals [4,5]. However, the effects of these herbs on dairy cows vary depending on the dose, types of nutrients and polyphenols, and their nature [6].

Plantain (*Plantago lanceolata* L.) is a protein-, vitamin-, and mineral-rich herb frequently utilized as a supplement in animals to produce milk, meat, and eggs [7–9]. It exerts antioxidants, anti-microbials, anti-parasitics, and anti-inflammatory effects on animal health, as it contains various classes of polyphenols such as acteoside, aucubin, and catalpol [10]. These polyphenols of plantain herb have been shown to improve milk yield and reduce urinary nitrogen flow and enteric methane emission in dairy cows, while increasing growth, immunity, and antioxidant levels in sheep [8,11,12]. Besides, it also encompasses β-carotene and polyphenols, which have a superior capacity to minimize OS [4], resulting in improving immunity, milk yield, and quality in dairy cows.

Lemongrass (*Cymbopogon citratus*) is broadly used in dairy production because it contains higher concentrations of carbohydrates, polyphenols, and zinc [13]. Feeding lemongrass to beef cattle (100 gm/day) and ewe (5 to 10 gm/day) positively alter the rumen microbiome and volatile fatty acid concentrations, thus resulting in an improvement of productivity and milk component yield [13–15], while feeding lemongrass to beef cattle and ewe causes inconsistent results on digestibility, immunity, and methane emission [16,17]. Besides, citral, a major polyphenol in lemongrass, acts as a precursor for the synthesis of β-carotene in animals. β-carotene and zinc act as potent antioxidants, which may improve milk yield, quality, and immunity by suppressing the OS in dairy cows. With these capabilities of lemongrass, it may improve immunity, serum and milk mineral concentrations, and milk yield in dairy cows.

Though both herbs are rich in polyphenols, vitamins, and minerals, to the best of our knowledge, no study has yet been done to evaluate the milk yield, immunity, liver enzymatic activity, and serum and milk mineral status in dairy cows offered dried plantain and/or lemongrass.

Moreover, this study examined how dried plantain and/or lemongrass powder supplementation influence milk yield, immunity, liver enzymes, serum, and milk mineral status in dairy cows.

## Materials and Methods

### Ethical approval

The entire experimental methodology was approved by the Animal Welfare and Experimentation Ethics Committee of Bangladesh Agricultural University, Bangladesh (AWEEC/BAU/2021/59).

### Animals, diets, design, and management

A total of 20 multiparous dairy cows (Holstein crossbred) were arbitrarily given to four diets with five cows per diet on a farm bordering to the Bangladesh Agricultural University (BAU), Mymensingh, Bangladesh (location 24^o^71'22.7''N, 90^o^42'80.9"E). Each cow was partially adjusted in the individual tie-stall (40 square feet) face-out barn. Among the 63 days of the feeding trial, the adaptation and collection periods were 7 and 56 days, respectively. From the data of the adaptation period, milk yield (kg, mean ± standard error) was analyzed for each group (Control: 8.12 ± 0.50; Plantain: 8.53 ± 0.51; Lemongrass: 8.54 ± 0.50; Plantain-lemongrass: 8.23 ± 0.54, and *p* = 0.916) using one-way analysis of covariance (ANCOVA), where days in milking (DIM), parity number (PN), and body weight (BW) were taken as covariates. The average values (mean ± SD) of DIM, PN, and BW of all cows were 102 ± 35 days, 3.0 ± 1.0, and 432 ± 38 kg on the first day of the adaptation period, respectively. The first dietary group was the control diet (CL-D), which was formed up of 51.1% concentrates and 48.9% German grass (*Echinochloa polystachya*) with 14.93% crude protein (CP) and 10.96 MJ ME/Kg dry matter (DM). Daily, each cow was fed CL-D with 100 gm plantain powder, 100 gm lemongrass powder, and 50 gm plantain + 50 gm lemongrass powder and considered as plantain diet (PT-D), lemongrass diet (LG-D), and plantain-lemongrass diet (PL-D), respectively. Milk and blood samples were taken four times, each with a 14-day gap, throughout the 56-day collection period. The results were analyzed using repeated measure two-way ANCOVA with DIM, PN, and BW as covariates and the dietary groups, time, and their interaction as fixed factors. Before and after milking, the cows received concentrate feeds (0630 and 1530 h) and German grass (0900 and 1800 h) twice daily, respectively.

The grass was cultivated near the farm yard, while concentrate items were purchased from the local market. In addition, each cow received plantain, lemongrass, and their combination powder in a bowl in the morning, with uptake confirmed by inspection. Plantain and lemongrass were produced at the Shahjalal Animal Nutrition Field Laboratory in the BAU using proper agronomic techniques and harvested at 65 and 60 days, respectively. Both herbs were dried for 4–5 days in the shade using artificial ventilation to ensure optimal wilting. Each herb was thoroughly dried before being ground into 1 mm particles and stored at 27^o^C to avoid the growth of mold and fungus. The ingredients of CL-D and the nutrient composition of CL-D and both herbs are shown in Table 1. Besides, cows were hand-milked twice daily, between 7:00 and 16:00 h. During the experimental period, *ad libitum* access to clean, fresh water and a similar environment (27.0^o^C ± 3.0^o^C, 70.0% ± 3.0% relative humidity) was provided to all cows. Using a hosepipe, the cows and barn were cleaned twice daily. The cows received a subcutaneous injection of a broad-spectrum anthelminthic (Invermac Plus, G-live, Animal Science Ltd. Bangladesh, dose: 1.0 ml per 50 kg BW) 14 days before the trial.

### Record-keeping, samples collection, and preparation

The daily intake of German grass and concentrates was calculated by subtracting ort feed from the supplied feed. German grass was gathered and dried for ration formulation and sampling. Then, dried German grass and all concentrates were processed, and the ration ingredients were accurately weighed. The ration and both herb powders were kept at −20°C until analyzed. The daily yield of milk was weighed in the morning and afternoon. Then, a 100-ml (morning: afternoon = 6:4 on a weight basis) milk sample was collected in two falcon tubes with 50 ml in each on days 14, 28, 42, and 56 following the adaptation period. Using plastic syringes and a 19 gm needle, a 10 ml blood sample was taken from the jugular vein of cows after 4 h feeding on days 14, 28, 42, and 56. A 15 ml sterile falcon tube was used to collect the blood sample, and it was given 30 min to coagulate. The serum was then extracted using a centrifuge machine (Z 306, Hermle, Germany) at 3,000 × gm for 15 min and stored in eppendorf tubes at −20°C until analysis.

**Table 1.** Ingredient composition and nutritional elements of basal diet (CL-D) and supplemented herbs.

Attributes	% on DM basis		
	CL-D	Plantain herb	Lemongrass
Ingredients			
German grass	48.9	-	-
Wheat bran	25.4	-	-
Mustard oil cake	8.9	-	-
Broken rice	7.4	-	-
^1^Ready-Mix feed	7.2	-	-
Salt	1.6	-	-
Di-Calcium phosphate	0.6	-	-
Nutrient composition			
Dry matter	31.2	89.7	91.5
Organic matter	90.3	84.5	91.1
CP	14.9	15.1	5.4
Crude fiber	22.3	16.6	26.5
NDF	38.3	36.0	63.1
ADF	22.3	24.4	41.1
Ether extract	4.7	2.9	5.2
Ash	9.7	15.5	8.9
Calcium	0.70	0.11	0.04
Phosphorus	0.46	0.25	0.09
Zinc		0.04	0.12
^#^Metabolizable energy (MJ kg/DM)	10.96	9.02	9.65

^1^Ready-Mix feed = 89.5% DM, 19.3% CP, 6.7% crude fiber; ^#^ = calculated value

CL-D = control diet; CP = crude protein; NDF = neutral detergent fiber; ADF = acid detergent fiber.

### Chemical analysis

The proximate components of the ration, herbal powder including plantain and lemongrass, were determined in accordance with the AOAC [18]. By subtracting the ash percentage from 100, the organic matter content of all samples were calculated. The percentages of neutral detergent fiber (NDF) and acid detergent fiber (ADF) in all samples were calculated in accordance with Goering and Van Soest [19]. Ration, herbs, and dried milk samples were digested using 10 ml, 6 mol/l HCl after ash determination at 550°C for 5 h. The digested sample was gradually raised at 160°C–170°C until fumes of HClO_4_ appeared, using a locally made digester. The digested sample volume was increased to 25 ml by adding distilled water and used for the determination of calcium (Ca), phosphorus (P), and zinc. Ca and P in all samples were determined by spectrophotometry using a UV spectrophotometer (T60; PG Instruments, Lutterworth, Leicestershire, UK) in the Animal Nutrition Laboratory, whereas zinc content was measured using a flame atomic absorption spectrophotometer (Model no. AA-7000; SHIMADZU, Kyoto, Japan).

On 14, 28, 42, and 56 days, 100 ml of fresh milk samples were collected for every cow in the morning to determine raw milk composition. Milk samples were analyzed within 2 h of milking to determine milk total solids (TS), solids not-fat (SNF), lactose, fat, and minerals by the MilkoScan SLP-60 Milk Analyzer (MIA-SLP-60). A raw milk sample (0.5 ml) was taken in a Kjeldahl flask with a catalyzer mixer, then 20 ml H_2_SO_4_ was added, digested at 100°C, and finally titrated with 0.1 N HCl for the determination of protein levels [18].

The serum total protein and albumin amounts were measured using enzymatic kits (Human Company, USA), and the amount of globulin was determined by deducting albumin from the total protein. Serum immunoglobulins G (IgG) and M (IgM) were determined using bovine ELISA kits (Sigma-Aldrich, St. Louis, MO). The concentrations of Ca, P, and zinc in the serum were measured using a URIT-810 bio-analyzer, according to the manufacturer’s protocol (Human Company, USA). Serum aspartate aminotransaminase (AST) (Kat no. K753, Bio-vision, USA) and alanine aminotransaminase (ALT) (Kat no. C9033, Sigma Aldrich, Germany) concentrations were assessed with commercial enzymatic assay kits from Sigma Aldrich (Germany) and Bio-vision (USA) using spectrophotometric methods by following the manufacturer’s instructions, respectively. Serum alkaline phosphatase (ALP) was determined with a URIT-810 bioanalyzer using the manufacturer’s protocol (Linear, Spain).

### Data analyses

Each sample from each cow was collected four times, and each sample was examined in triplicate. MS Excel was used to manage the raw data afterward, and SPSS 22 was used to analyze it. The two-way repeated measure ANCOVA was used in the present study to determine the significant factors connected to the response variables. The empirical repeated measures ANCOVA model for each response variable is given below:

*Yij*= *αi* + *Di* +*Tj* +*DiTj* + β1DIM + β2PN + β3BW +*εij*;

*i, j*= 1, 2, 3, 4.

Where Y denotes the response variable; αi denotes the average value of the output taking into account the random effects of the cow; D, T, and DT represent diet, time, and the interaction of diet and time taken into consideration as fixed effects, respectively. DIM, PN, and BW stand for days in milking, parity number, and body weight respectively, each considered a covariate.

Four diets were used, and measurements were taken every 14 days on days 14, 28, 42, and 56. Multiple comparisons among the diet means were performed using the least significant difference method at *p* ≤ 0.05.

## Results

### DM intake, milk yield, and its composition of dairy cows

**Figure 1.**
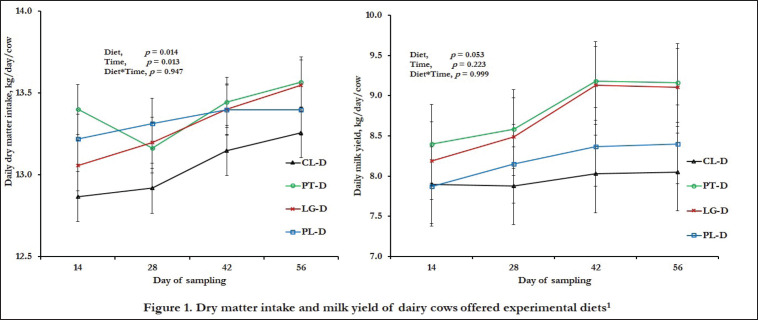
DM intake and milk yield of dairy cows offered experimental diets^1^. ^1^Experimental diets: CL-D = German grass and concentrate-based diet having CP 14.9% and ME 11 MJ/kg DM; PT-D = CL-D + 100 gm plantain powder/cow per day; LG-D = CL-D + 100 gm lemongrass powder/cow per day; PL-D = CL-D + 50 gm plantain and 50 gm lemongrass powder/cow per day.

Dairy cows consumed more DM (p < 0.05), and showed the propensity of improvement in milk yield (p = 0.053) as time went on, while herbal supplements (PT-D, LG-D, and PL-D) enhanced overall DM consumption and milk yield by 2%–3% and 3%–11% compared to CL-D, respectively (p ≤ 0.05) (Fig. 1). However, the milk yield of PL-D showed a better value but was comparable to CL-D (*p* > 0.05). Moreover, LG-D improved the composition of TS, SNF, protein, and fat compared to the CL-D group, while the lowest fat and SNF were obtained in PT-D (*p <* 0.05) (Table 2). Besides, except TS, other milk components were alike with increasing time (*p* > 0.05), while the interaction of herbal supplements and time untouched all milk components (*p* > 0.05).

### Serum protein indices, immune status, and liver enzyme activity of dairy cows

**Figure 2.**
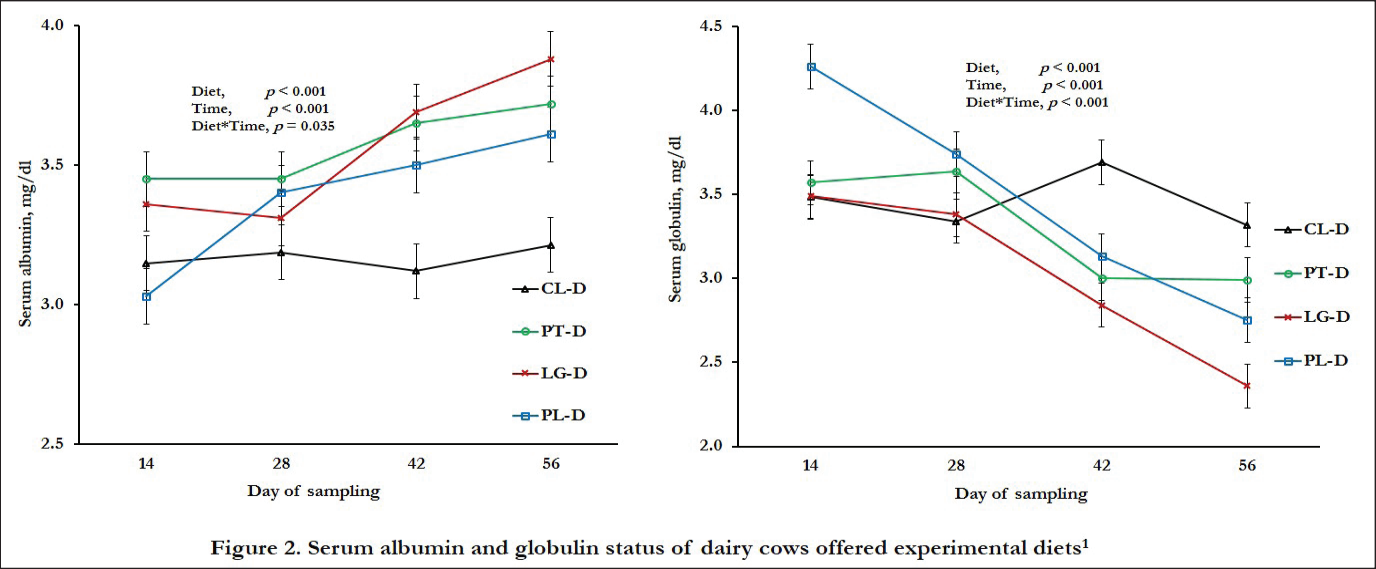
Serum albumin and globulin status of dairy cows offered experimental diets^1^. ^1^Experimental diets: CL-D = German grass and concentrate-based diet having CP 14.9% and ME 11 MJ/kg DM; PT-D = CL-D + 100 gm plantain powder/cow per day; LG-D = CL-D + 100 gm lemongrass powder/cow per day; PL-D = CL-D + 50 gm plantain and 50 gm lemongrass powder/cow per day.

Herbal supplements, time, and their interactions increased blood albumin levels but decreased serum globulin concentrations (*p* < 0.05) (Fig. 2). Herbal supplements considerably elevated serum albumin (7%–13%) and reduced serum globulin (5%–13%) (*p* < 0.05), though PL–D and CL–D had comparable levels of globulin (*p* > 0.05). Besides, the serum total protein of cows displayed variable values in herbal-supplemented groups, while greater serum IgG and IgM concentrations were obtained in herbal supplements compared to CL-D (*p <* 0.01) (Table 3). Compared to the CL-D, herbal supplementation had a beneficial effect on liver enzymes, i.e., lower serum AST, ALT, and ALP concentrations (*p* < 0.01), while the lowest ALT and ALP values were obtained in the PT-D.

### Serum and milk minerals status of dairy cows

Herbal supplementation showed no influence on serum and milk Ca and P levels (*p >* 0.05) when compared to the CL-D, although it improved serum zinc concentrations (*p <* 0.01) (Table 4). In addition, the LG-D group had the highest serum (+7%) and milk (+2%) zinc concentrations than other groups (*p <* 0.01). Besides, time and its interaction with herbal supplements had a substantial impact on serum zinc concentrations (*p* < 0.05), while serum and milk Ca and P levels, and milk zinc exhibited inconsistent effects (*p* > 0.05).

**Table 2.** Milk composition of dairy cows offered experimental diets.

Variables (%)	Experimental diets^1^				SEM	*p-value* ^2^		
	CL-D	PT-D	LG-D	PL-D		D	T	D × T
Total solids	12.01^bc^	11.75^c^	12.48^a^	12.19^ab^	0.07	0.01	0.02	0.65
Solids-not-fat	8.10^b^	8.15^b^	8.49^a^	8.48^a^	0.04	<0.01	0.07	0.10
Lactose	4.08	4.10	4.24	4.19	0.03	0.16	0.20	0.16
Protein	3.29^b^	3.30^b^	3.55^a^	3.51^a^	0.03	0.01	0.59	0.08
Fat	3.95^ab^	3.61^c^	3.99^a^	3.71^bc^	0.04	0.01	0.07	0.88

^a–c^Means within a row with different superscripts differ (*p <* 0.05*)* for the interaction between experimental diets and time.

^1^Experimental diets: CL-D = German grass and concentrate-based diet having CP 14.9% and ME 11 MJ/kg DM; PT-D = CL-D + 100 gm plantain powder/cow per day; LG-D = CL-D + 100 gm lemongrass powder/cow per day; PL-D = CL-D + 50 gm plantain and 50 gm lemongrass powder/cow per day.

^2^Probability of treatment effects: D = effects of experimental diets; T = effect of time; D × T = interaction between experimental diets and time.

**Table 3.** Serum protein, immune and liver enzymes activity of dairy cows offered experimental diets.

Variables	Experimental diets^1^				SEM	*p* value^2^		
	CL-D	PT-D	LG-D	PL-D		D	T	D × T
Serum protein indices and immune status (mg/dl)								
Total protein	6.69^ab^	6.75^a^	6.57^b^	6.86^a^	0.03	0.01	<0.01	<0.01
IgG	15.37^c^	18.84^b^	21.48^a^	21.26^a^	0.16	<0.01	0.02	0.81
IgM	2.44^c^	2.80^a^	2.67^ab^	2.82^a^	0.03	<0.01	0.06	0.49
Liver health status (IU/l)								
AST	40.94^a^	33.17^b^	32.12^b^	31.86^b^	0.35	<0.01	<0.01	<0.01
ALT	16.94^a^	8.13^c^	9.05^bc^	10.64^b^	0.39	<0.01	<0.01	0.02
ALP	83.74^a^	53.45^d^	77.00^b^	74.25^c^	0.34	<0.01	<0.01	<0.01

^a–d^Means within a row with different superscripts differ (*p <* 0.05) for the interaction between experimental diets and time.

^1^Experimental diets: CL-D = German grass and concentrate-based diet having CP 14.9% and ME 11 MJ/kg DM; PT-D = CL-D + 100 gm plantain powder/cow per day; LG-D = CL-D + 100 gm lemongrass powder/cow per day; PL-D = CL-D + 50 gm plantain and 50 gm lemongrass powder/cow per day.

^2^Probability of treatment effects: D = effects of experimental diets; T = effect of time; D × T = interaction between experimental diets and time.

IgG = immunoglobulin G; IgM = immunoglobulin M; AST = aspartate aminotransferase; ALT = alanine aminotransferase; ALP = alkaline phosphatase.

**Table 4.** Serum and milk minerals status of dairy cows offered experimental diets.

Variables	Experimental diets^1^				SEM	*p value* ^2^			
	CL-D	PT-D	LG-D	PL-D		D	T	D × T	
Serum minerals									
Zinc (µg/dl)	99.35^d^	101.08^c^	106.64^a^	104.54^b^	0.19	<0.01	<0.01		0.01
Calcium (mg/dl)	8.11	8.46	8.06	8.20	0.06	0.09	<0.01		0.81
Phosphorus (mg/dl)	4.38	4.63	4.51	4.46	0.04	0.15	0.34		<0.01
Milk minerals (mg/kg)									
Zinc	3.82^c^	3.83^c^	3.90^a^	3.86^b^	<0.01	<0.01	0.32		0.42
Calcium	1.49	1.49	1.49	1.48	<0.01	0.64	0.01		0.25
Phosphorus	1.02	1.03	1.03	1.03	<0.01	0.05	0.16		0.90

^a–d^Means within a row with different superscripts differ (*p <* 0.05) for the interaction between experimental diets and time.

^1^Experimental diets: CL-D = German grass and concentrate-based diet having CP 14.9% and ME 11 MJ/kg DM; PT-D = CL-D + 100 gm plantain powder/cow per day; LG-D = CL-D + 100 gm lemongrass powder/cow per day; PL-D = CL-D + 50 gm plantain and 50 gm lemongrass powder/cow per day.

^2^Probability of treatment effects: D = effects of experimental diets; T = effect of time; D × T = interaction between experimental diets and time.

## Discussions

Elevated DM intake was observed in cows that were given both plantain and lemongrass herbs as a supplement. However, feeding high or low levels of plantain through pasture unaffected DM consumption in dairy cows [11,12,20]. Besides, lemongrass at 5 or 10 gm per day for ewes [13] and 100, 200, and 300 gm for beef cattle [14] unaffected DM consumption. This might be due to ruminants receiving inappropriate doses of these herbs for their health and production [6]. Plantain with basal rations improved DM uptake in dairy cows [7] and sheep [8,21], while lemongrass at 4 gm per day increased DM intake in Burki goats [22]. Both findings are consistent with the current study. This result might be attributed to their antioxidant, antimicrobial, hepatoprotective, and anti-inflammatory activities, which trigger the release of salivary, gastric, and pancreatic enzymes while accelerating the activity of pancreatic lipase and amylase [23]. Besides, both herbs positively influenced rumen microbes and nutrient digestibility, thus resulting in greater DM consumption in cows. This greater DM intake resulted in a better milk yield (10%–11%) in cows given plantain and lemongrass. Aligned to the current study, dairy cows grazed swards or forb with 23.0% [7] and 18.4% [20] plantain on a DM basis, and dairy ewes [13] and goats [22] given 5%–10% by weight lemongrass powder substantially improved milk yield by 7%–27%. Besides, both herbs contain polyphenols, which may reduce ROS formation and promote dairy cow health, increasing milk yield. Better liver enzymes, albumin, and serum immunoglobulin might boost milk yield in cows [24]. Due to better immunity and liver health, PL-D increased the milk yield compared to CL-D but not significantly. A blend of plantain-lemongrass polyphenols may reduce rumen-beneficial bacteria [15], thus resulting in lower milk yield in PL-D compared to PT-D and LG-D. Cows given LG-D had more milk fat and protein. This may be attributed to the elevated fiber digestibility and acetate levels in cows raising milk fat percent [13], while lemongrass also transports polyphenols and zinc to milk via serum, thus improving milk protein levels [25].

Other studies [7,16] revealed that serum total protein levels in cows varied among herb supplementation groups. However, in the current study, cows given herbs boosted serum albumin and lowered globulin concentrations owing to the herbs’ anti-inflammatory activity [24]. The anti-inflammatory properties of limonene in lemongrass and aucubin in plantain may have increased serum IgG and IgM in our dairy cows [8,26]. Furthermore, earlier studies [8,21,27] revealed that plantain and commercial acteoside substantially lowered serum liver enzyme (ALT, AST, and ALP) concentrations in ruminants, which is consistent with the current study. Lemongrass had no impact on serum liver enzymes in Holstein steers [16], while limonene and citral administered to rats and mice significantly reduced them [28]. Herbal supplementation may improve cow liver health because aucubin in plantain, and citral and limonene in lemongrass improve mitochondrial activity by increasing peroxisome proliferator-activated receptor gamma coactivator-1 and uncoupling protein-2 expression and messenger ribonucleic acid expression by enhancing cytochrome P450 enzymes and reducing OS and apoptosis in rats [28,29]. Plantain and lemongrass failed to improve the serum and milk Ca and P levels due to their low doses and their uptake for bone metabolism [30,31]. Dairy cows fed additionally 59 mg of zinc daily raised milk zinc levels by 3.0% [32], whereas a daily supplement of 41 mg of additional zinc via lemongrass enhanced milk zinc concentrations by 2.09% in LG-D. Similar to previous studies [33,34], the current study showed that daily LG-D and PL-D containing 119 and 80 mg zinc improved milk zinc by 2.09% and 1.05%, respectively.

## Conclusion

Both plantain and lemongrass improved DM intake, immunity, liver health, and milk yield, while their combination enhanced immunity, liver health, and serum and milk zinc concentrations in dairy cows. Besides, lemongrass boosted serum and milk zinc concentrations. However, supplementation with plantain and/or lemongrass did not influence serum and milk calcium and phosphorus levels in dairy cows. Due to the abundance of polyphenols in both herbs, additional research on dairy cows‘ serum and milk antioxidant levels and ability to digest nutrients may be explored.

## List of Abbreviations

ADF, acid detergent fiber; ALP, alkaline phosphatase; ALT, alanine aminotransaminase; AST, aspartate aminotransaminase; ANCOVA, analysis of covariance; BW, body weight; CL-D, control diet; CP, crude protein; DIM, days in milking; DM, dry matter; ELISA, enzyme-linked immunosorbent assay; gm, grams; h, hours; IgG, immunoglobulin-G; IgM, immunoglobulin-M; IU, international unit; l, liter; Kg, kilograms; LG-D, lemongrass diet; ME, metabolizable energy; mg, milligram; min, minutes; MJ, megajoule; ml, milliliter; N, normal; NDF, neutral detergent fiber; OS, oxidative stress; PN, parity number; PL-D, plantain-lemongrass diet; PT-D, plantain diet; ROS, reactive oxygen species; SD., standard deviation; SEM, standard error of mean; SNF, solids not-fat; TS, total solids; β, beta; µg, microgram.
